# Unmasking the “Braided” Bowel: Chronic Intestinal Pseudo-Obstruction—A Case Report

**DOI:** 10.3390/diagnostics16121762

**Published:** 2026-06-08

**Authors:** Chun-Heng Hung, Chung-Ta Lee, Po-Chuan Chen

**Affiliations:** 1 Department of Surgery, National Cheng Kung University Hospital, College of Medicine, National Cheng Kung University, Tainan 704302, Taiwan; genehung2003@gmail.com; 2 Department of Pathology, National Cheng Kung University Hospital, College of Medicine, National Cheng Kung University, Tainan 704302, Taiwan; lcta@mail.ncku.edu.tw; 3 Division of Colorectal Surgery, Department of Surgery, National Cheng Kung University Hospital, College of Medicine, National Cheng Kung University, Tainan 704302, Taiwan

**Keywords:** chronic intestinal pseudo-obstruction, gastrointestinal dysmotility, acute abdomen, surgical pathology

## Abstract

**Background and Clinical Significance:** Chronic intestinal pseudo-obstruction (CIPO) clinically presents as a mechanical obstruction. When patients present with signs of impending ischemia, emergency exploratory laparotomy is indicated. Differentiating CIPO from other etiologies preoperatively is highly challenging, making intraoperative findings and definitive histopathology crucial for an accurate diagnosis. This report aims to elucidate a novel macroscopic presentation of myopathic CIPO and explore the potential biomechanical mechanisms underlying the observed structural anomaly. **Case Presentation:** A 37-year-old male with no prior abdominal surgery presented with a high-grade bowel obstruction and early signs of ischemia. Emergency laparotomy revealed a unique “braided dough twist” anomaly of the distal small bowel, prompting a segmental resection. Subsequent histopathological and immunohistochemical evaluations revealed severe outer longitudinal muscle atrophy with an intact enteric nervous system, confirming myopathic CIPO. Following surgery, the patient recovered uneventfully with significant nutritional improvement. **Conclusions:** Myopathic CIPO can manifest as a striking “braided” bowel anomaly—a macroscopic presentation previously undocumented in the literature. This case illustrates that determining the optimal extent of resection relies heavily on intraoperative visual and tactile assessment, and that comprehensive histopathological profiling is essential for establishing a definitive diagnosis and elucidating the underlying biomechanical mechanisms of this unique structural anomaly.

## 1. Introduction

Chronic intestinal pseudo-obstruction (CIPO) is a rare disorder characterized by severe impairment of gastrointestinal propulsion. Clinically, it presents as a mechanical obstruction in the absence of any anatomical occluding lesions [1,2]. Based on the underlying neuromuscular defect, CIPO is categorized into three subtypes: neuropathic, myopathic, and mesenchymopathic (involving the interstitial cells of Cajal) [3,4]. Due to its non-specific clinical presentation and the lack of specific early biomarkers, the diagnosis of CIPO is often delayed. Patients often experience years of recurrent, unexplained obstructive symptoms before a correct diagnosis is established [5].

In acute settings, preoperatively distinguishing CIPO from other causes of bowel obstruction remains highly challenging. Computed tomography (CT) typically demonstrates severe intestinal dilation and stasis, confirming the presence of a high-grade obstruction. However, these findings are non-specific and lack the discriminatory power to differentiate an anatomical occluding lesion from a functional etiology such as CIPO [5]. As a result, surgeons frequently perform emergency laparotomy for acute bowel obstruction, with CIPO rarely considered the underlying diagnosis preoperatively [6]. During these surgical explorations, surgeons may encounter variable macroscopic findings such as segmental dilatation, thickening or thinning of the bowel wall, or abnormal bowel configuration [1,7]. However, these gross intraoperative findings are neither specific nor pathognomonic for CIPO, let alone sufficient to distinguish between its neuropathic, myopathic, or mesenchymopathic subtypes [4]. Therefore, accurate diagnosis and subtype categorization of CIPO necessitates full-thickness specimens and comprehensive immunohistochemical profiling following standardized guidelines such as the London Classification [3].

We report a case of an adult male presenting with high-grade mechanical obstruction, in whom emergency laparotomy revealed a unique ‘braided’ macroscopic bowel anomaly ultimately diagnosed as myopathic CIPO. This report aims to highlight the diagnostic challenges of myopathic CIPO in an acute setting. We emphasize the importance of recognizing distinct macroscopic and tactile features, and integrating them with comprehensive immunohistochemistry to establish a definitive diagnosis, while also elucidating the potential biomechanical mechanisms underlying this unique ‘braided’ configuration.

## 2. Case Presentation

A 37-year-old male with no prior abdominal surgery presented to the emergency department with a 3-day history of progressive abdominal distension, severe pain, nausea, and bilious vomiting. His medical history included intermittent abdominal pain since early adulthood, without a definitive diagnosis. On physical examination, the abdomen was markedly distended and diffusely tender, with peritoneal signs including involuntary muscle guarding and rebound tenderness. Laboratory tests revealed hemoconcentration (hemoglobin 16.8 g/dL) and mild hypokalemia (3.4 mmol/L). Additional markers indicated chronic malnutrition, including low prealbumin (11.9 mg/dL), transferrin (133.0 mg/dL), and zinc (22 μg/dL). The serum lactate level was mildly elevated at 2.48 mmol/L with a normal pH (7.39). These findings, combined with peritonism, suggested impending bowel ischemia.

Abdominal computed tomography (CT) demonstrated a high-grade mechanical small bowel obstruction. Axial views showed a transition between the dilated proximal loops in the right hemiabdomen and the collapsed distal loops in the left hemiabdomen. The “small-bowel feces sign” was identified within the dilated loop immediately proximal to the transition point (Figure 1). Given the clinical presentation, peritoneal signs, and laboratory evidence of impending ischemia, an emergency exploratory laparotomy was performed.

Intraoperatively, the distal small bowel exhibited a tightly coiled “braided dough twist” configuration fixed by dense adhesions (Figure 2). This macroscopic anomaly contrasted with the proximal dilated loops, which displayed paper-thin, translucent walls and patchy serosal adhesions (Figure 3). Careful exploration revealed a visual and tactile transition along the affected bowel, progressing from a tactilely normal jejunum to a paper-thin and structurally compromised proximal ileum, and ultimately terminating in the braided distal segment. To relieve the obstruction and mitigate ischemic risks, a segmental resection was performed. Guided by these tactile and visual cues, the resection encompassed not only the most dysfunctional “braided” portion but also a segment of the proximally dilated bowel. The resection was carefully extended proximally until a relatively normal intestinal segment was reached, facilitating a safe primary anastomosis.

A definitive diagnosis was established through histopathological evaluation of the full-thickness resected specimen, following the London Classification guidelines. Microscopic examination revealed severe atrophy and a near-total absence of the outer longitudinal layer of the muscularis propria, while the inner circular layer remained relatively intact (Figure 4A). Masson’s trichrome staining demonstrated the replacement of the muscular defect with dense collagen fibers (Figure 4B). Immunohistochemical (IHC) analysis demonstrated an intact enteric nervous system and a normal population of interstitial cells of Cajal (CD117 positive), excluding enteric neuropathy or mesenchymopathy (Figure 5). Microscopic evaluation of the serosa revealed interloop adhesions (Figure 6). These adhesions were present without significant fibrosis of the underlying bowel wall, suggesting that they were secondary to chronic stasis and the macroscopic configuration, rather than a primary inflammatory process. These findings confirmed the diagnosis of myopathic CIPO.

Following resection and primary anastomosis, the patient recovered without complications, with complete resolution of his acute obstructive symptoms and successful resumption of oral intake. Postoperatively, metoclopramide was initiated at 10 mg three times daily before meals as a promotility agent and subsequently discontinued as bowel function normalized. Given the chronic and relapsing nature of CIPO, a long-term surveillance plan has been established: nutritional parameters—including prealbumin, transferrin, and micronutrients—will be reassessed every three months during the first postoperative year and semi-annually thereafter, with abdominal imaging performed on a symptom-driven basis or upon clinical deterioration. The patient has also been referred for genetic counseling clinic for further evaluation and guidance. At the 6-month follow-up, he remained asymptomatic and off all medications, with significant weight gain (from 63 kg to 70 kg) and normalization of his nutritional markers. The patient has continued regular outpatient follow-up without evidence of recurrence.

## 3. Discussion

The present case exemplifies the diagnostic and surgical challenges of myopathic CIPO presenting acutely, while introducing a macroscopic bowel configuration not previously described in this condition. The following discussion addresses three aspects of this case: the diagnostic trajectory from inconclusive imaging to definitive pathological subtyping, the intraoperative findings and decision-making that guided a successful targeted resection, and the proposed biomechanical mechanisms underlying the formation of this unprecedented ‘braided’ morphology.

The initial clinical and radiological workup in our patient illustrates how profoundly CIPO can present as mechanical obstruction. The disease often follows a gradual ‘acute-on-chronic’ course [8], and our patient’s longstanding history of recurrent abdominal pain since adulthood reflecting years of unrecognized dysmotility before this acute exacerbation. Diagnostic imaging performed during this acute episode revealed the “small-bowel feces sign” (SBFS); however, SBFS is a nonspecific marker of profound intestinal stasis that can equally arise from severe dysmotility, and is not pathognomonic for mechanical obstruction—thereby limiting the ability of radiological findings alone to distinguish the precise etiology [1,9]. Accordingly, the preoperative differential diagnosis remained broad: closed-loop small bowel volvulus and internal hernia were considered, as both can produce a similar CT appearance of a transition zone with proximal dilatation and distal collapse. Chronic inflammatory processes—including Crohn’s disease—were also entertained given the presence of dense interloop adhesions in a young male without a prior diagnosis. In this case, the onset of peritonism and elevated serum lactate, signaling impending bowel ischemia, necessitated an emergency laparotomy. Intraoperatively, these alternative diagnoses were systematically excluded. The absence of mesenteric twisting, identifiable rotation point, or hernial orifice ruled out volvulus and internal hernia. Furthermore, the lack of transmural thickening, skip lesions, or creeping fat excluded inflammatory bowel disease. Most importantly, laparotomy not only revealed the unprecedented ‘braided’ macroscopic configuration but also secured the full-thickness tissue required to definitively diagnose myopathic CIPO.

Historically, surgical intervention in CIPO has been viewed with great caution. Previous studies have demonstrated that surgical management in CIPO patients is frequently associated with high postoperative morbidity and a high likelihood of re-operation. In the largest published surgical cohort of adult CIPO patients, overall morbidity reached 58.2% with a mortality rate of 7.9%, and re-operation probabilities of 44%, 60%, and 66% at 1, 3, and 5 years, respectively [10]. These poor outcomes are primarily attributable to the pan-enteric nature of the underlying dysmotility and the chronic malnutrition invariably present in these patients [1]. Nevertheless, a recent scoping review of surgical interventions for CIPO suggests that targeted surgical intervention in carefully selected patients with localized structural complications may still be safe and effective, although strong conclusions remain limited by the small sample sizes of existing studies [11]. While our patient’s preoperative nutritional markers reflected chronic malabsorption—a well-recognized risk factor for poor wound healing and anastomotic failure—the favorable postoperative course in this case supports this selective approach. The key to this outcome lay in recognizing the distinct visual and tactile features intraoperatively—namely, the transition from a tactilely normal jejunum to a paper-thin proximal ileum, terminating in the braided distal segment. These findings prompted a targeted segmental resection of the dysfunctional loop rather than simple adhesiolysis. While the underlying dysmotility may affect the gastrointestinal tract more broadly [12,13], eliminating the most severely dysfunctional segments and anastomosing to a relatively healthy intestinal segment reduced the burden of the dysmotility. This approach not only mitigated the risk of postoperative leakage associated with diseased CIPO bowel but also led to the resolution of the patient’s obstructive symptoms and normalization of his nutritional status at the 6-month follow-up.

The full-thickness specimens obtained during resection enabled a comprehensive histopathological workup, ultimately establishing the etiology of this unique CIPO presentation. Because CIPO encompasses three underlying phenotypes—neuropathic, myopathic, and mesenchymopathic—differentiating among them cannot be achieved clinically or radiologically; it relies on advanced pathological evaluation [14,15,16]. Previous studies have highlighted the limitations of conventional pathology in these scenarios. Standard histology fails to detect neuromuscular abnormalities in almost one-third of CIPO cases, as routine H&E staining frequently misses subtle pathological changes. In a cohort of 21 adult CIPO patients, systematic immunohistochemistry using Hu C/D detected hypoganglionosis in 29% of patients—four of whom were undiagnosed on standard histology—while CD117 (c-kit) identified interstitial cells of Cajal (ICC) defects in 48% of patients, including two with no histologic structural abnormality on routine examination [4]. Therefore, a comprehensive immunohistochemical panel is necessary to distinguish these subtypes: neuropathic forms exhibit anomalies in the enteric neural plexus detectable with neuronal markers such as Hu C/D, Bcl-2, and S100 protein, while mesenchymopathic forms are characterized by a depletion of CD117-positive ICC [3].

Following the London Classification guidelines, IHC profiling of our full-thickness specimen demonstrated an intact enteric nervous system and a preserved ICC network (CD117 positive). These findings ruled out enteric neuropathy and mesenchymopathy. Masson’s trichrome staining demonstrated the severe, selective atrophy of the outer longitudinal muscle layer and its extensive replacement by dense collagenous fibrosis. Full-thickness evaluation further confirmed the absence of transmural inflammation, granulomata, and mucosal architectural distortion, pathologically excluding Crohn’s disease and other chronic inflammatory processes. This pathological differentiation not only confirmed a pure myopathic CIPO but also offered a potential biomechanical explanation for the patient’s profound failure of intestinal propulsion. Recent translational research has shed further light on the tissue-level mechanisms underlying longitudinal muscle atrophy in CIPO. A 2026 morphometric study by Boschetti et al. demonstrated that idiopathic CIPO is characterized by significantly higher intestinal fibrosis, selective thinning of the longitudinal muscle layer, and vascular remodeling—findings consistent with our histopathological observations, particularly regarding the longitudinal muscle atrophy and fibrotic replacement [17]. Critically, these structural alterations were accompanied by a downregulation of angiogenic mediators, including thymidine phosphorylase and VEGF, alongside upregulation of HIF-1α, indicating a state of chronic intestinal hypoxia. Furthermore, multivariate analysis in that study linked reduced vascular area and longitudinal muscle thinning directly to higher frequency of sub-occlusive episodes, providing a mechanistic framework that connects microvascular dysfunction to progressive neuromuscular degeneration and disease severity in CIPO. Although our case demonstrated preserved enteric neurons (Figure 5A)—differing from the neuronal loss observed in that cohort—the shared features of longitudinal muscle atrophy and fibrosis suggest that the near-total longitudinal muscle loss observed in our patient may represent the end-stage consequence of this chronic vascular and fibrotic remodeling process, albeit with a predominantly myopathic rather than mixed neuromyopathic subtype.

A comprehensive review of the medical literature reveals that myopathic CIPO generally falls into two broad categories. The first encompasses generalized, nonspecific dilations: published intraoperative and radiological descriptions consistently report diffusely or segmentally dilated, atonic, and flaccid bowel loops, often accompanied by serosal thinning, discoloration, or prominent air-fluid levels [1,5,7,18,19,20]. Radiologically, these classic presentations are characterized by the absence of a distinct transition point and the lack of a true mechanical obstruction. The second category comprises specific congenital developmental defects tightly linked to severe syndromic phenotypes—such as the microcolon and megacystis characteristic of megacystis-microcolon-intestinal hypoperistalsis syndrome (MMIHS), the widespread smooth muscle failure seen in multisystemic smooth muscle dysfunction syndrome (MSMDS), and the anatomical aberrations associated with congenital short bowel syndrome (CSBS) and hydrocephalus with stenosis of the aqueduct of Sylvius (HSAS) [13,21]. Beyond these two categories, unusual macroscopic morphologies have occasionally been documented. To objectively position our patient’s unique findings within this historical spectrum, we conducted a structured literature search across the PubMed and Embase databases from inception to March 2026, with the search strategy and results compiled in Supplementary Table S1. This structured review confirmed that a primary, fixed ‘braided’ small bowel configuration in a pristine abdomen has never been reported, thereby substantiating the absolute macroscopic novelty of this case. We postulate that the “braided” configuration observed in our patient represents a distinct, acquired morphological change—one driven not by early developmental arrest, but by chronic biomechanical stress and dysregulated smooth muscle contractions accumulating over decades of disease progression.

As demonstrated by our full-thickness pathology, the near-total loss of the longitudinal muscle layer fundamentally disrupts the biomechanical equilibrium of the intestine. Normally, the longitudinal and circular muscle layers contract in a coordinated manner during peristalsis, with essential force transmission occurring between the neighboring smooth muscle layers [12]. Previous studies have shown that selective atrophy or fibrosis of the longitudinal muscle layer is a recognized pathologic feature of myopathic CIPO where the outer longitudinal layer is often more severely affected than the inner circular layer in adult patients [17,22]. In myopathic CIPO, this smooth muscle structural defect characteristically manifests on intestinal manometry as low-amplitude contractions with decreased frequency, a pattern distinct from neuropathic forms which typically preserve contraction amplitude [15,23]. Although manometry was not performed in our patient due to the acute surgical presentation, the histopathological findings of near-total longitudinal muscle loss with fibrotic replacement are consistent with this manometric pattern. Biomechanical studies demonstrate that the intestinal wall exhibits different stress–strain properties in the longitudinal versus circumferential directions, and longitudinal stretch significantly affects circumferential stress distribution [24,25]. We hypothesize that without the longitudinal layer to facilitate local longitudinal shortening and counteract circumferential contraction forces, this profound mechanical imbalance may have driven the bowel to abnormally elongate and ultimately twist into its unique “braided” configuration—a macroscopic finding not previously reported in the literature.

Following the formation of this “braided” configuration, a secondary mechanism may account for its irreversible fixation. As shown in our microscopic evaluation (Figure 6), interloop adhesions were present specifically within the narrowed and elongated braided segment, notably without significant fibrosis between the adhered serosal surfaces. We hypothesize that this transition from a dynamic twisting to a fixed anatomical deformity may have been facilitated by sustained apposition and structural crowding within the complexly coiled loops. This unique configuration brought adjacent serosal surfaces into sustained, tight apposition. Experimental studies have demonstrated that mesothelial cells respond to mechanical stimuli by undergoing mesothelial-to-mesenchymal transition (MMT), a process driven by mechanotransduction pathways including YAP activation [26]. Furthermore, in vitro models have shown that mesothelial cells can generate expansive membrane protrusions that tether opposing surfaces with substantial adherence forces, a process that precedes matrix deposition and is triggered by calcium signaling and cytoskeletal effectors [27]. Damage to mesothelial cells, regardless of the inciting stimulus, is recognized as the initiating event for adhesion formation [28], and this process can occur in the context of sterile injury without primary transmural inflammation [29]. These interloop adhesions, acting as a physical restraint, may have stabilized the distorted loops into a fixed “braided” configuration. While the longitudinal muscle layer itself underwent extensive fibrotic replacement (Figure 4B), the relative paucity of dense inter-serosal fibrosis suggests these adhesions represent a dynamic remodeling response to structural entanglement rather than a static fibrotic scar (Figure 6).

Once the “braided” configuration is stabilized, it functions as a fixed, irreversible mechanical obstruction. The accumulation of gas and fluid proximal to the obstruction leads to bowel distension and increased intraluminal pressure [30]. According to Laplace’s law, wall tension is proportional to intraluminal pressure and radius, and inversely proportional to wall thickness [31,32,33,34]. Bowel dilatation increases mural tension and decreases mural tensile strength, thereby increasing perforation risk [35]. At this transition zone, the intestinal wall—already compromised by the loss of longitudinal muscle support—undergoes extreme biomechanical compensation. In typical intestinal obstruction, experimental studies demonstrate that the bowel wall responds to increased pressure through adaptive remodeling, including wall thickening and increased tissue stiffness, with the most pronounced changes occurring in the segment immediately proximal to the obstruction [36,37]. However, in our case, the near-total absence of the longitudinal muscle layer precludes this normal compensatory hypertrophy. Instead of adaptive thickening, the structurally deficient wall undergoes progressive thinning (Figure 3), representing a point of structural vulnerability at the interface of the obstruction.

Notably, this thinning exhibits a distinct spatial gradient; as we move further upstream from the “braided” anchor, the bowel wall appears relatively healthy despite remaining dilated. Studies on partial intestinal obstruction have shown that morphomechanical changes occur throughout the proximal bowel, characteristically manifesting as adaptive wall thickening, with the most pronounced alterations observed in the segment immediately proximal to the obstruction site [36]. We speculate that in our patient, this spatial distribution of remodeling may be inverted: rather than maximal hypertrophy at the obstruction interface, the absence of longitudinal muscle results in maximal thinning at this site, where biomechanical stress is most concentrated. Consequently, the more proximal segments, despite being dilated, retain relatively greater structural integrity compared to the extreme wall thinning observed at the frontline of the braided obstruction. This spatial gradient may be explained by the distribution of intraluminal pressure: while the accumulation of gas and fluid proximal to an obstruction leads to bowel distension and increased intraluminal pressure, this pressure is highest at the immediate transition zone and dissipates as the stasis volume increases further upstream, thereby reducing the biomechanical stress on more proximal segments [30,31].

The selective loss of the longitudinal muscle layer—the defining pathological feature in our case—raises the possibility of an underlying smooth muscle cytoskeletal abnormality; however, the precise pathogenesis remains uncertain in the absence of molecular testing. *ACTG2* mutations have been identified as the most common genetic cause of visceral myopathy, manifesting clinically across a broad phenotypic spectrum: ranging from megacystis-microcolon-intestinal hypoperistalsis syndrome (MMIHS) at the severe end to late-onset visceral myopathy [38,39]. Notably, *ACTG2*-associated visceral myopathy has been reported to manifest with diverse anatomical abnormalities, including chronic intestinal pseudo-obstruction, intestinal malrotation, hypertrophic pyloric stenosis, and choledochal cyst [40]. One recent study has further confirmed that pathogenic *ACTG2* mutations were identified in all myopathic cases of pediatric intestinal pseudo-obstruction, underscoring the central role of this gene in myopathic CIPO [15].

The clinical significance of this genetic classification extends beyond diagnosis to prognosis and surgical decision-making. In the largest adult CIPO cohort reported to date—a 2026 study comprising 130 patients with a median follow-up of 18 years—integrated genetic and histological evaluation characterized 82% of patients and stratified them into distinct histogenetic subgroups [41]. Patients with monogenic myopathy, predominantly driven by *ACTG2* and *MYH11* mutations, demonstrated the most favorable long-term survival (adjusted hazard ratio: 0.06; *p* = 0.004) and the highest rates of clinical improvement following bowel resection—83% after enterectomy and 77% after colectomy—compared to other subgroups. Although genetic testing was not performed in our patient due to financial constraints, the observed histopathological findings may share certain features with previously reported monogenic visceral myopathies.

At the molecular level, *ACTG2* encodes enteric smooth muscle actin gamma-2, a protein essential for contraction of smooth muscles in the intestines and bladder. Pathogenic variants disrupt actin filament polymerization through multiple mechanisms, including premature protein degradation, impaired interactions with actin-binding proteins, inhibited polymerization, and filament destabilization [42]. Immunohistochemical studies have demonstrated that intestinal smooth muscle from patients with *ACTG2* variants shows reduced levels of cytoplasmic ACTG2 and abnormal accumulation of the protein into intracellular inclusions [43]. In a cohort study of 53 families with visceral myopathy, a molecular diagnostic rate of 64% was reported, of which 97% was attributed to *ACTG2* [44]. These previously described molecular abnormalities may offer one hypothetical framework for interpreting the selective smooth muscle atrophy observed in our case. The near-total loss of the longitudinal muscle layer with fibrotic replacement may reflect a distinct pathogenic mechanism or a more severe variant. Alternatively, it may represent the cumulative structural consequence of decades of progressive disease. The unprecedented “braided” configuration and extreme wall thinning observed in our case further suggest that distinct macroscopic findings during surgical exploration may represent a distinctive morphological pattern that warrants further clinicopathological and genetic investigation in similar cases.

## 4. Conclusions

Myopathic CIPO can manifest as an acute mechanical obstruction, posing substantial diagnostic challenges when conventional clinical and radiological assessments are non-specific. The “braided” bowel configuration presented here represents a previously undocumented macroscopic presentation, offering surgeons a distinctive visual and tactile landmark to guide the optimal extent of resection—a judgment that imaging alone cannot provide. The resulting full-thickness specimens, analyzed with comprehensive immunohistochemical profiling following standardized guidelines such as the London Classification, are then indispensable for establishing a definitive diagnosis and elucidating the biomechanical basis of this unique structural presentation. Accurate diagnosis of this complex disorder hinges on the integration of intraoperative findings with comprehensive pathological evaluation. Although genetic testing was not performed in the present case because of financial constraints, future incorporation of genetic counseling and molecular testing in similar cases of suspected myopathic CIPO may help clarify the underlying pathogenesis and broaden current understanding of this rare entity.

## Figures and Tables

**Figure 1:**
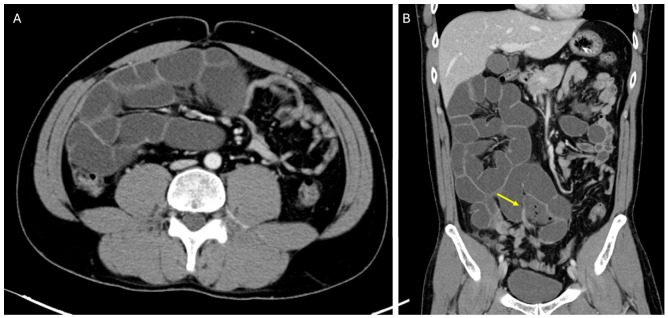
Abdominal computed tomography. (**A**) Axial view demonstrating a marked discrepancy in bowel caliber, with dilated proximal small bowel loops occupying the right hemiabdomen and collapsed loops in the left hemiabdomen. (**B**) Coronal view showing a transitional zone. The “small-bowel feces sign” is noted immediately proximal to the apparent obstruction point (yellow arrow).

**Figure 2:**
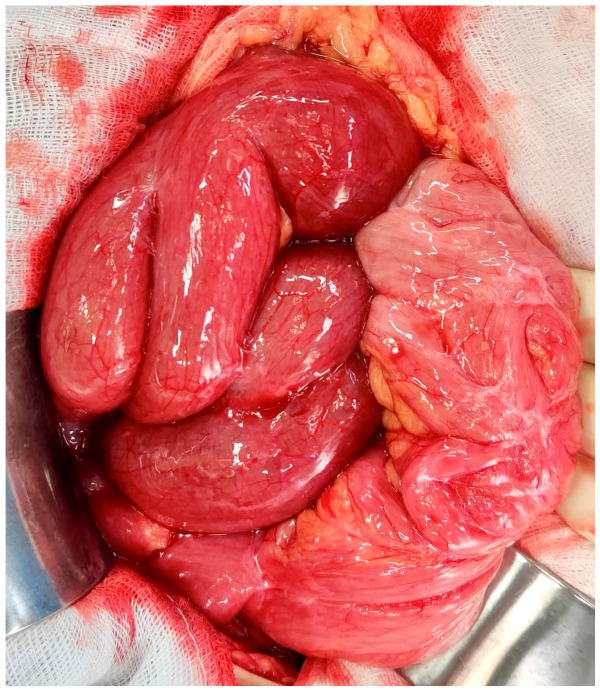
Intraoperative view. The right hemiabdomen revealed dilated proximal small bowel loops, corresponding to the preoperative computed tomography findings. The distal small bowel in the left hemiabdomen—which appeared as collapsed loops on the CT images—was found to be fixed in a highly unusual “braided dough twist” configuration.

**Figure 3:**
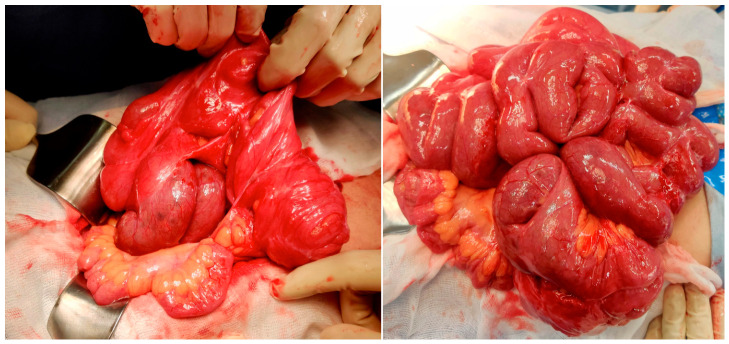
Intraoperative view. Upstream dilation secondary to the distal obstruction. The affected bowel segments exhibit patchy serosal adhesions.

**Figure 4:**
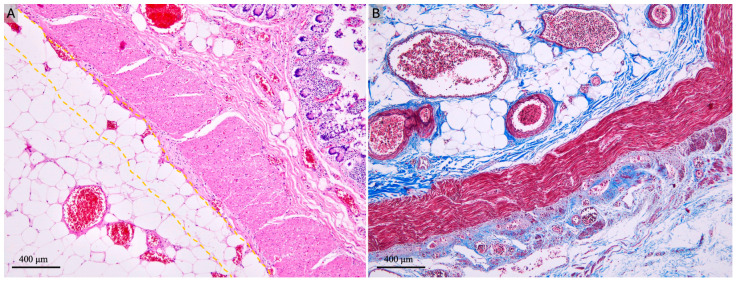
Histopathological evaluation of the full-thickness specimen (Scale bar = 400 μm for both panels). (**A**) Microscopic examination reveals a relatively intact inner circular muscle layer with severe atrophy and near-total absence of the outer longitudinal layer. The orange line indicates the expected anatomical territory of the missing outer layer. (**B**) Masson’s trichrome staining highlights the replacement of the longitudinal muscle defect with dense collagen fibers (stained blue).

**Figure 5:**
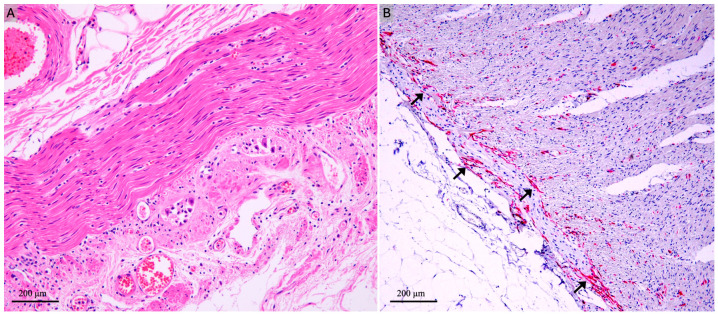
Histopathological evaluation of the enteric nervous system and interstitial cells of Cajal (Scale bar = 200 μm for both panels). (**A**) Microscopic examination demonstrates an intact enteric nervous system with normal neurons. (**B**) Positive c-kit (CD117) staining highlights a preserved network of interstitial cells of Cajal, visible as spindle-shaped, red-stained cells (Black arrows indicate representative cells).

**Figure 6:**
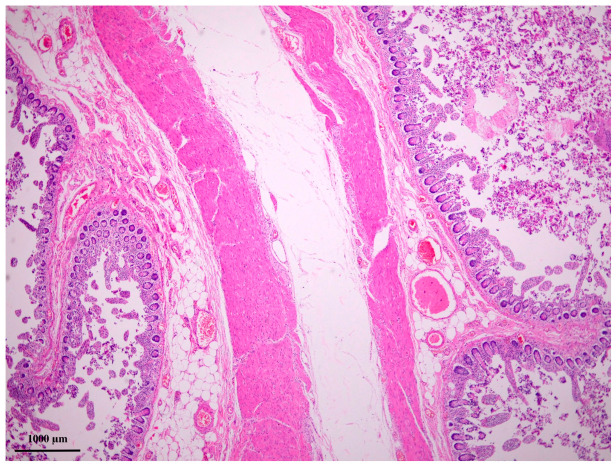
Histopathological evaluation of the intestinal serosa (Scale bar = 1000 μm). Microscopic examination demonstrates interloop adhesions with an absence of significant fibrosis in the underlying bowel wall.

## Data Availability

The data presented in this study are available on request from the corresponding author due to privacy or ethical restrictions.

## Supplementary Materials

The following supporting information can be downloaded at: https://www.mdpi.com/article/10.3390/diagnostics16121762/s1, Table S1: Macroscopic Findings in Published Cases of CIPO and Related Visceral Myopathies. References [45,46,47,48,49,50,51,52,53,54,55,56,57,58] are cited in the supplementary materials.

## Abbreviations

The following abbreviations are used in this manuscript:

CIPO	Chronic intestinal pseudo-obstruction
CT	Computed tomography
SBFS	Small-bowel feces sign
H&E	Hematoxylin and eosin
ICC	Interstitial cells of Cajal
IHC	Immunohistochemical
MMIHS	Megacystis-microcolon-intestinal hypoperistalsis syndrome
MSMDS	Multisystemic smooth muscle dysfunction syndrome
CSBS	Congenital short bowel syndrome
HSAS	Hydrocephalus with stenosis of the aqueduct of Sylvius
ACTG2	Actin gamma-2
MYH11	Myosin heavy chain 11
VEGF	Vascular endothelial growth factor
HIF-1α	Hypoxia-inducible factor 1-alpha
YAP	Yes-associated protein
MMT	Mesothelial-to-mesenchymal transition
