# Supplementary material for: Unmasking the “Braided” Bowel: Chronic Intestinal Pseudo-Obstruction—A Case Report
Source: Diagnostics (Basel). 2026 Jun 8;16(12):1762. doi: 10.3390/diagnostics16121762 (PMC13298276; doi:10.3390/diagnostics16121762)
Supplement: Supplementary file 1 [file diagnostics-16-01762-s001.zip › diagnostics-4241350-Supplementary.pdf]

Supplementary Table S1. Macroscopic Findings in Published Cases of CIPO and Related Visceral Myopathies

A structured literature search of the PubMed and Embase databases (inception to March 2026) was performed using the following search string: ("chronic intestinal pseudo-obstruction" OR "CIPO" OR "visceral myopathy") AND ("intraoperative findings" OR "laparotomy" OR "macroscopic appearance" OR "bowel configuration" OR "twisted bowel" OR "coiled bowel" OR "braided bowel" OR "volvulus" OR "adhesions"). Included literature encompassed any relevant studies that contained explicit intraoperative macroscopic descriptions.

Study (Year)	CIPO Subtype	N	Age / Sex	Macroscopic Findings	Structural Configuration Noted
Schuffler et al. (1977) Gastroenterology [45]	Myopathic	1	15F	Small bowel and colon diffusely dilated with no mechanical obstruction. Subsequent laparotomies consistently showed diffuse dilatation.	Diffuse dilatation
Anuras et al. (1979) Ann Surg [46]	Myopathic	7	Variable	Megaduodenum, dilated distal jejunum and proximal ileum. Adhesions in two patients postoperatively.	Segmental dilatation; Sigmoid volvulus in one case
Schuffler & Deitch (1980) Ann Surg [47]	Mixed (Myopathic & Neuropathic)	15	Variable	All cases: Segmental or diffuse dilatation of the GI tract without mechanical obstruction (exhibiting distinct clinicoanatomic patterns).<br>Case 8: Small intestine initially 'thought to be rotated on itself,' but re-exploration confirmed a massively dilated jejunum with multiple diverticula and no mechanical obstruction.<br>Case 9 (Miscellaneous): Concomitant incarcerated umbilical hernia and small/large bowel volvulus (noted as a disease-related clinical complication due to bowel redundancy, rather than a surgical complication).	Diffuse/segmental dilatation ± redundancy; secondary volvulus in one case
Mitros et al. (1982) Hum Pathol [48]	Myopathic	14	Variable	Segmental dilatation of the alimentary tract, often involving multiple sites, with megaduodenum being the most common feature. I<br>Patient 6: Megaduodenum and dilated loops of small bowel with adhesions were explicitly noted.	Segmental dilatation and thinning; adhesions in one patient
Darvishian & Basham (2002) Ann Clin Lab Sci [49]	Myopathic	1	35M (autopsy)	Autopsy: Massive distension of the large and small intestines, accompanied by a dilated and thickened esophagus. Severe fibroneoplastic interloop adhesions were noted throughout the abdominal cavity, attributable to concomitant disseminated signet-ring cell carcinomatosis.	Massive diffuse dilatation; adhesions from concomitant carcinomatosis
Granero Castro et al. (2010) Int Arch Med [50]	Myopathic (MNGIE)	2	22F, 27F	Case 1: Intraoperative finding of a massively dilated colon; subtotal colectomy specimen with loss of colonic haustra.<br>Case 2: Intraoperative finding of a perforated duodenal diverticulum.	Diffuse colonic dilatation with loss of haustras and duodenal diverticula
Burcharth et al. (2011) Case Rep Surg [51]	Myopathic	1	89F	Perforated cecum with faecal peritoneal soilage. No intraabdominal tumor or mechanical obstruction identified intraoperatively. In distended areas, the bowel wall is thinned to approximately 1 mm with intact mucosa.	Diffuse segmental dilatation of the colonic loops
Nakajima et al. (2012) Pathol Int [52]	Myopathic	1	50F	Midgut volvulus secondary to intestinal malrotation was observed. Gross examination of the resected specimen revealed a diffusely dilated and dark red intestine, with gradual tapering of the cut-end margins to a normal diameter.	Secondary midgut volvulus due to intestinal malrotation
Chaffin et al. (2016) Appl Immunohistochem Mol Morphol [22]	Myopathic	2	44M, 67M	Case 1 (67M): Redundant sigmoid colon specimen with a grossly thinned outer longitudinal layer of the muscularis propria.<br>Case 2 (44M): Small bowel specimen measuring 8.5 cm in length and 3.0 cm in diameter, showing grossly appreciable necrosis.	Redundant colon with adhesions; diffuse dilated small bowel

Wrenn et al. (2017)<br>Int J Surg Case Rep [53]	Myopathic	1	60M	Entire colon extremely dilated and redundant; sigmoid mesocolon elongated with a narrow base. Gross examination of the resected colon specimen demonstrated extreme thinning of the bowel wall throughout.	Diffuse colonic dilatation and redundancy with sigmoid mesocolon elongation
Lim et al. (2020)<br>Cureus [54]	Unspecified	1	68M	Massive dilatation of the entire small bowel (up to 15 cm) with pneumatosis intestinalis and gas bubbles within the mesentery; no mechanical obstruction or perforation site identified.	Diffuse small bowel dilatation with pneumatosis
Shi et al. (2021)<br>BMJ Case Rep [55]	Myopathic	1	51M	A perforation in the mid-distal jejunum with localized thickened and friable bowel wall near the segment. Minimal intraabdominal adhesions were encountered despite the patient's history of multiple prior abdominal surgeries.	Jejunal perforation; minimal adhesions
Underwood et al. (2021)<br>BMJ Case Rep [56]	Myopathic (MIDD)	1	33M	Distended small bowel with no transition point; turbid peritoneal fluid with no macroscopic source of contamination.	Diffuse small bowel dilatation; no transition point
Kim et al. (2023)<br>Am Surg [57]	Neuropathic	1	72M	Diffuse colonic dilatation and edema, most prominent in the descending and sigmoid colon, with transmural sigmoid ischemia.	Diffuse colonic dilatation with ischemia
Palmisani et al. (2026)<br>Int J Colorectal Dis [58]	Myopathic (ADL)	1	11F	Caecal volvulus. Histology showed 'tiger-striped' fibrosis and atrophy of muscular layers.	Secondary caecal volvulus in ADL; characteristic 'tiger-striped' fibrotic pattern on histology
**PRESENT CASE (2026)**	**Myopathic**	**1**	**37M**	**Transition from tactilely normal jejunum to paper-thin proximal ileum terminating in a segmental 'braided dough twist' configuration of the distal ileum. Multiple loops interdigitated and fixed by dense interloop adhesions. No prior abdominal surgery.**	**Fixed, segmental braided configuration**

Abbreviations: CIPO = chronic intestinal pseudo-obstruction; MNGIE = mitochondrial neurogastrointestinal encephalomyopathy; MIDD = maternally inherited diabetes and deafness; ADL = African degenerative leiomyopathy; N = number of patients.

Note: The present case (highlighted) is the only reported instance of a primary, fixed, multi-loop 'braided' small bowel configuration as a direct macroscopic manifestation of myopathic CIPO in a patient without prior abdominal surgery, distinguishing it from the secondary volvulus and adhesion-related structural changes observed in previously reported cases.
